# Fiber-based quantum-dot pulse oximetry for wearable health monitoring with high wavelength selectivity and photoplethysmogram sensitivity

**DOI:** 10.1038/s41528-023-00248-1

**Published:** 2023-03-17

**Authors:** Ho Seung Lee, Byeongju Noh, Seong Uk Kong, Yong Ha Hwang, Ha-Eun Cho, Yongmin Jeon, Kyung Cheol Choi

**Affiliations:** 1 School of Electrical Engineering, Korea Advanced Institute of Science and Technology (KAIST), Daejeon, Republic of Korea; 2 Department of Biomedical Engineering, Gachon University, Seongnam, Republic of Korea

**Keywords:** Electrical and electronic engineering, Applied optics, Quantum dots, Biosensors

## Abstract

Increasing demand for real-time healthcare monitoring is leading to advances in thin and flexible optoelectronic device-based wearable pulse oximetry. Most previous studies have used OLEDs for this purpose, but did not consider the side effects of broad full-width half-maximum (FWHM) characteristics and single substrates. In this study, we performed SpO_2_ measurement using a fiber-based quantum-dot pulse oximetry (FQPO) system capable of mass production with a transferable encapsulation technique, and a narrow FWHM of about 30 nm. Based on analyses we determined that uniform angular narrow FWHM-based light sources are important for accurate SpO_2_ measurements through multi-layer structures and human skin tissues. The FQPO was shown to have improved photoplethysmogram (PPG) signal sensitivity with no waveguide-mode noise signal, as is typically generated when using a single substrate (30–50%). We successfully demonstrate improved SpO_2_ measurement accuracy as well as all-in-one clothing-type pulse oximetry with FQPO.

## Introduction

At the beginning of the 4th industrial revolution, there is increasing interest in next-generation wearable devices capable of providing real-time healthcare monitoring^1–3^. Because they can be carried close to the body, such devices enable diagnosis and prevention as well as treatment. Among various real-time diagnostic methods, a number of bio-application studies are investigating wearable optoelectronic devices, which provide non-invasive advantages by using light. The recent COVID-19 pandemic has also increased interest in optoelectronic-based wearable pulse oximetry, which can help identify hypoxia caused by lung disease^4–6^.

Pulse oximetry monitors information about oxyhemoglobin and deoxyhemoglobin based on a photoplethysmogram (PPG), using two light sources with different wavelengths, and allows users to check their peripheral oxygen saturation levels (SpO_2_) based on a calibration process. Using non-invasive light, it is not only safe, simple, convenient and cheap but also capable of rapidly checking SpO_2_^7–9^. With these advantages, pulse oximetry are widely used in hospitals and have attracted attention for next-generation wearable healthcare. However, existing pulse oximetry systems mainly use a point light source LED-based transmission method, and are bulky and difficult to carry as well as inflexible, so have been primarily used for the treatment and diagnosis of patients in hospitals.

Recently, with the increase in demand for real-time health diagnosis and prevention, and the development of the next-generation health care industry, there has been increasing research on reflection-type pulse oximetry. Using thin and flexible wearable optoelectronic devices, this approach would allow health monitoring anywhere on the human body. In particular, studies are being conducted to develop advanced transmission-type pulse oximetry suitable for use exclusively on fingers and earlobes^10^.

Organic light-emitting diodes (OLEDs) are organic thin film-based electro-luminescence (EL) devices, typically several hundred nano-meters thick, and suitable for use in many wearable applications because of their very light weight and flexible characteristics^11–13^. Similar studies have also been published on quantum-dot light-emitting diodes (QDLEDs), which are based on a thin emissive layer composed of quantum-dots (QDs)^14,15^. However, because the organic layer is vulnerable to moisture, there are some reliability limitations in medical applications. Lately, there have also been reports on washable encapsulation techniques which can enhance the reliability of wearable optoelectronic devices for medical applications^16–18^.

Many previous studies about wearable pulse oximetry have mainly focused on the form-factor advantages of OLEDs^19^. Recent research has also reported improvements such as lower power^20^, higher accuracy^10^, free driving power^21^ and self-powered pulse oximetry, in addition to the form-factor advantage^22^ which are significant positive trends for wearable pulse oximetry. However, their application is often limited by the structure of patch-type wearable pulse oximetry, which is commonly implemented on a 2-dimensional (2D) substrate.

To realize practical devices such as clothes that a person can actually wear, and to solve the lack of flexibility and breathability of existing 2D substrates, pulse oximetry is being implemented on a 1D fiber that can be woven into a 3D-substrate^23,24^. Recent studies on thin film EL devices based on fiber optoelectronics have been reported^25,26^. However, these have simply focused on the form-factor, without conducting bio-medical application studies.

In this study, we demonstrate a fiber-based quantum-dot pulse oximetry (FQPO) device with narrow full-width half-maximum (FWHM) characteristics, that has high measurement accuracy and can be woven into actual wearable clothes. The proposed FQPO achieves reduced process tact time and cost by simultaneously manufacturing multi-fibers using a roll-to-roll process. Its high measurement accuracy is based on its uniform wavelength selectivity. Even in harsh environments involving interference, absorption, and scattering, and different angles of the device on skin, it showed narrow FWHM characteristics of about 30 nm and lambertian characteristics. Ultimately, we succeeded in maximizing the accuracy of the reflection-type pulse oximetry device by introducing a fiber substrate that can be woven into a 3D form. This minimizes the waveguide mode-based direct current (DC) noise of vital signals produced by a single 2D substrate. The study results confirm the feasibility of a clothing-type pulse oximetry application using this FQPO.

## Results and discussion

### Design and concept of the proposed FQPO

**Fig. 1:**
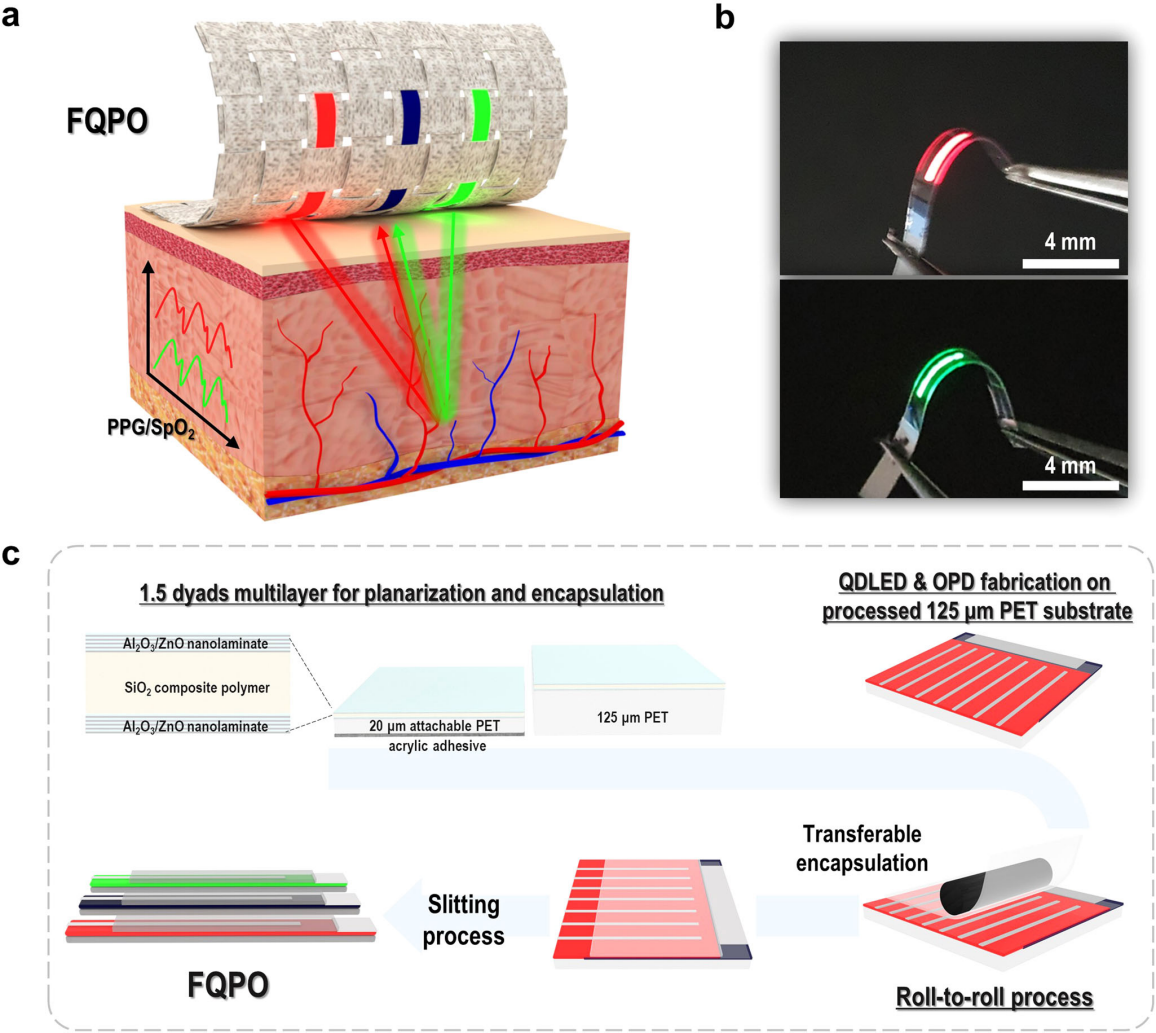
FQPO concept and fabrication. **a** Concept and schematic illustration of the FQPO. **b** The photo of the flexible red and green FQDLEDs which are emitting. **c** The fabrication process of the FQPO using the transferable encapsulation technique-based on 20-µm-thick attachable PET film.

Figure 1a shows a schematic illustration of the FQPO concept with the intended goal of use in clothing, and the measurement of a PPG signal and SpO_2_. A pulse oximetry basically requires two light sources and a sensor to monitor vital signals. For a weavable all-in one clothing type FQPO, the devices were designed in the form of fibers. SpO_2_ is obtained through a calibration process based on the PPG signal from the two light sources. For this purpose, a red light source with a relatively high molar extinction coefficient of deoxihemoglobin compared to oxyhemoglobin, and a NIR or green light source having the opposite characteristics, are often used. In the present study, red and green QD materials with narrow FWHM characteristics were used to increase wavelength selectivity, compared to the previously reported 2D substrate-based pulse oximetry, which is important for SpO_2_ measurement. In addition, flexible organic photodiodes (OPDs) that enable vital signal monitoring were applied. Then, a reflection-type pulse oximetry was fabricated as a versatile FQPO-based SpO_2_ monitoring device with aesthetics and convenience (Fig. 1a).

Figure 1b contains a photo of the flexible red and green FQDLED, and Fig. 1c shows the FQPO fabrication process using transferable encapsulation for mass production. Details of the QDLED and OPD structures and fabrication methods are provided in Methods Section. The FQPO was manufactured to be simple, uncomplicated and robust by utilizing a flexible and transparent polyethylene terephthalate (PET) substrate and Poly(3,4-ethylenedioxythiophene)-poly(styrenesulfonate) (PEDOT:PSS), which is a transparent conductive electrode (TCE)^27^. Since the QDLED and OPD are vulnerable to water and oxygen, it is very important to use a flexible thin film encapsulation process that is flexible and has superior water vapor transmission rate (WVTR) properties, to prevent the penetration of water and oxygen.

Figure 1c shows the fabrication of reliable fiber-based optical devices using a roll-to-roll process with an ultra-thin transferable encapsulation of 20 µm. Transferable encapsulation was implemented through a 1.5 dyads inorganic/organic multibarrier. The multibarrier was composed of an alumina/zinc oxide (Al_2_O_3_/ZnO, 3 nm/3 nm) nanolaminate-based 30 nm-thick inorganic film for superior WVTR properties, and for flexibility a silica (SiO_2_) composite polymer which was sandwiched as an inorganic nanolaminate layer^28^. In this way, the top and bottom encapsulation process can be carried out at the same time, and the process tact time and cost can be reduced^17^. Then, a slitting process was performed to complete the final FQDLED, and the optical characteristics and reliability were compared. (Supplementary Fig. 1). The slitting process technology is very important for fabricating a thin and reliable FQDLED fiber^29^. Laser cutting technologies for polymers such as PET have advanced a lot^30^, and if it can be additionally grafted, a thinner and more reliable FQDLED can be produced.

The proposed fiber-based optical device manufacturing process can be applied to existing simple, fast, and widely used processes such as spin-coating, thermal evaporation, and atomic layer deposition (ALD) using a flat substrate. It has the advantage of allowing the mass-production of reliable wearable optical devices.

In this study, we demonstrate PPG measurement and SpO_2_ monitoring using the FQPO concept shown in Fig. 1a. To realize a high-performance wearable pulse oximetry, we analyzed the advantages of the FQPO with wavelength selectivity, a narrow FWHM and high PPG sensitivity in fiber-based optical devices.

### Optoelectrical characteristics and reliability of the flexible TCE-based FQDLED

**Fig. 2:**
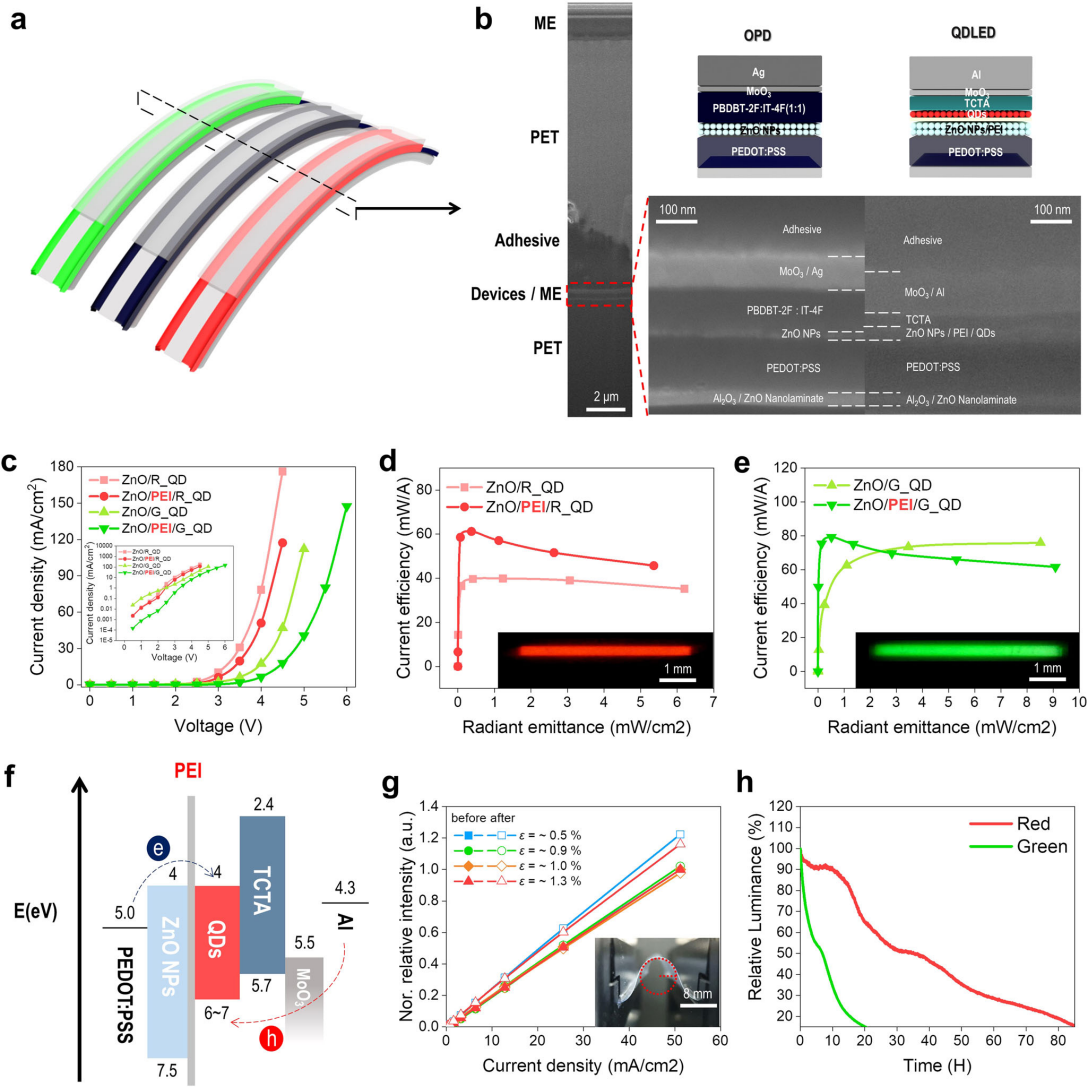
Cross-section images of the fiber-based devics and the characteristics of the red and green FQDLEDs. **a** Schematic illustration of the flexible red and green FQDLEDs and FOPD. **b** Cross-sectional SEM image and the device structures of the FQDLED and FOPD. **c** Current density-Voltage (J–V) characteristics according to the PEI insulating layer of the red and green FQDLEDs. **d**, **e** Comparison of the current efficiency with or without the PEI insulating layer of the red and green FQDLEDs. **f** Schematic energy diagram of the PEI insulating layer-based inverted QDLED. **g** Normalized relative intensity-current density characteristics with bending test of the FQDLED according to strain (1000 times repetition each). **h** Lifetime of the red FQDLED (LT20)/green FQDLED (LT15) with constant current operating (initial power consumption: 0.18 mW).

Figure 2a is a schematic illustration of the flexible FQPO and Fig. 2b shows the focused ion beam (FIB) cross-sectional image of the entire FQDLED. It confirms that a thin film has been uniformly formed. To realize a flexible FQPO, a flexible and transparent PEDOT:PSS electrode was used (Supplementary Fig. 2a). It has a sheet resistance of about 100 Ω/□ at 135 nm-thickness and a transmittance of about 83–89% in the visible range, which mean that it has appropriate performance as a cathode electrode in the inverted QDLED (Supplementary Fig. 2b, 2c, Supplementary Table 1). The PEDOT:PSS electrode also has extremely flexible characteristics. There was no change in sheet resistance after bending 1000 times, even up to a strain of about 4.17%, compared to an indium-tin-oxide (ITO) electrode, which increased in sheet resistance even at a strain of about 0.63% (Supplementary Fig. 3a–d).

**Fig. 3:**
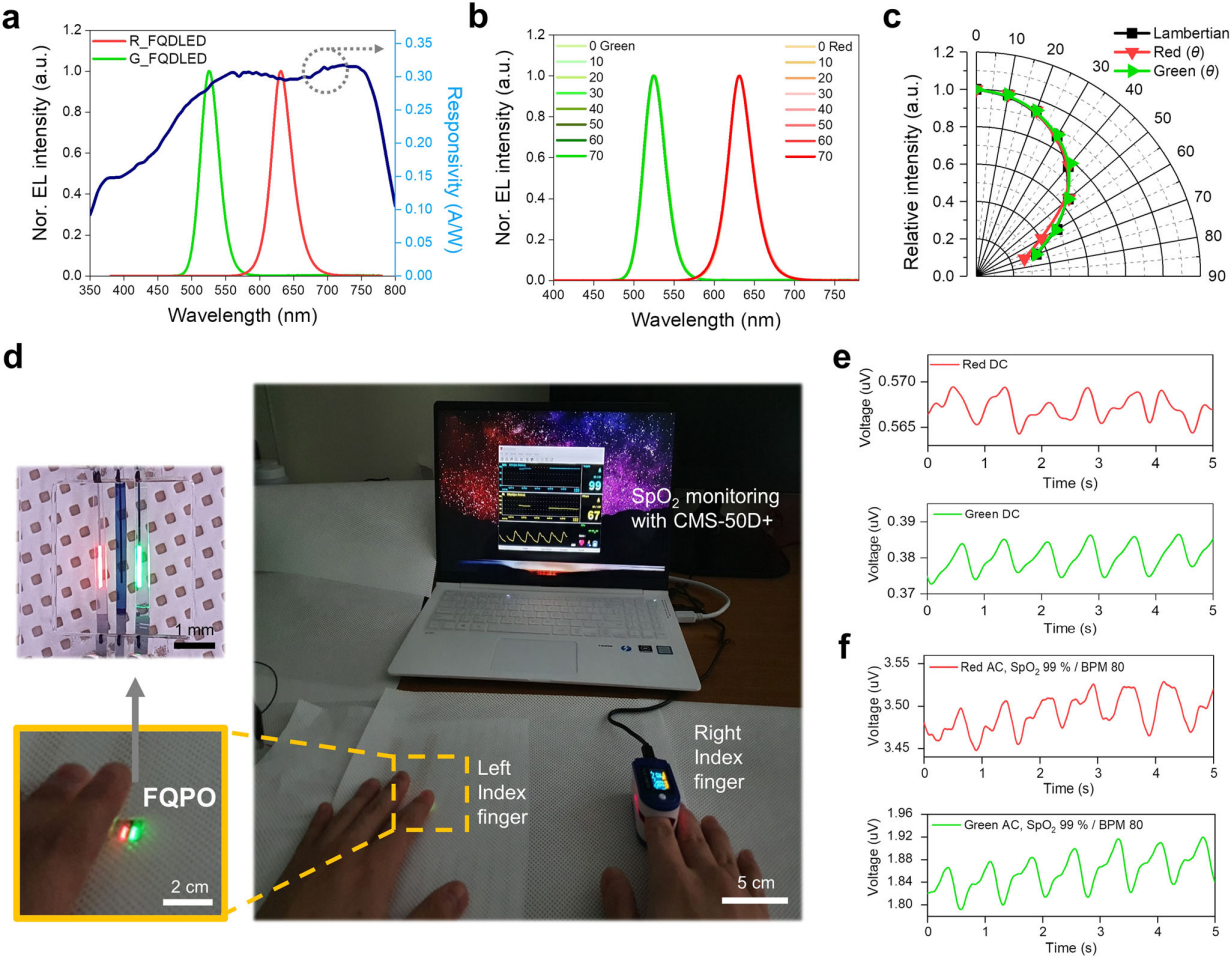
Spectral characteristics and SpO_2_ monitoring with the FQPO. **a** Normalized EL intensity and responsivity characteristics per wavelength of the FQPO. **b** Uniform angular spectral characteristics of the red and green FQDLEDs. **c** Angular relative EL intensity of the red and green FQDLEDs with lambertian characteristics. **d** Pictures of the left-right index fingers-based SpO_2_ monitoring. For SpO_2_ monitoring with the FQPO, measurement of the PPG signal from the left index finger with the FQPO and SpO_2_ monitoring from the right index finger with CMS-50D+, which is the commercial pulse oximetry device, were conducted simultaneously. **e** PPG signal with direct current (DC) driving of the FQPO (Power consumption: red 0.03 mW/green 0.031 mW). **f** SpO_2_ monitoring with alternating current(AC) driving of the FQPO (10 Hz, 30% duty cycle, power consumption: red 0.69 mW/green 0.91 mW, SpO_2_ 99%, BPM 80).

In this study, inverted structure-based devices were adopted that used ZnO nanoparticles (NPs) as the electron transport layer (ETL) on top of the PEDOT:PSS layer. This is consistent with previous studies that used ZnO NPs coated on PEDOT:PSS, where highly efficient optical devices were obtained regardless of the acidity characteristics of the PEDOT:PSS^24–26,31^. An FQDLED is an electron-dominant device based on an inverted QDLED, using ZnO NPs as the ETL. There have been numerous reports on the charge balance of inverted QDLEDs using the electron injection suppression method^32,33^. Here, a polyethylenimine (PEI) insulating layer was used to suppress electron injection. When the PEI was applied, the current efficiency was increased by suppressing electron injection (Fig. 2c–f). With the PEI insulating layer, the highest current efficiency was 61.22 mW/A for red and 79.28 mW/A for green (Fig. 2d, e, Table 1.). Notably, the FQPO succeeded with a PPG signal measurement at a low radiant emittance of about 0.1 mW/cm^2^. Notably, a maximum current efficiency was achieved in low light power using the PEI insulating layer (Fig. 3e).

**Table 1:** FQDLED & FOPD characteristics.

	Peak $\lambda$ (nm)	FWHM (nm)	V_th_ (V)	Max current efficiency (mW/A)	Responsivity (A/W)	D^*^_human_ (Jones)
Red FQDLED	631	34	~2	61.22	0.298	7.01 × 10^**10**^
Green FQDLED	526	32	~2.6	79.28	0.291	6.84 × 10^**10**^

In Supplementary Fig. 4, using the electron only device, the current efficiency was increased by the electron injection suppression mechanism with the PEI insulating layer. At this time, the current density was proportional to the voltage at the beginning of the current injection, and when the injection voltage was increased, the current density was proportional to 1.5 times the power voltage. This shows the analysis was reliable by following the space-charge-limited current (SCLC) mechanism during normal operation of the QDLED.

To evaluate the reliability of the FQDLED, bending tests and operating lifetime tests were performed. In the FQDLED, the neutral axis was quite close to the QDLED device with 20 µm-thickness PET of transferable encapsulation film (Supplementary Fig. 3e). The results showed it was very flexible up to a strain of 1.3% after being bent 1000 times (Fig. 2g). The FQDLED had less flexibility compared to the PEDOT:PSS electrode, and this can be attributed to the strain limitation of the multi-barrier, which contains inorganic layers with high Young’s Modulus.

In addition, the FQDLED showed a long operating reliability of up to 80 h (LT20, Red FQDLED) and 20 h (LT15, Green FQDLED) under air exposure (Fig. 2h). Lifetime was measured at power consumptions from 0.18 mW to about 0.03 mW, which was the lowest power consumption where a PPG signal could be measured (Fig. 3e, Supplementary Fig. 5). Based on this, the lifetime of the FQPO was determined using the Green FQDLED. This is because a non-fullerene-based OPD has a very stable lifetime of more than 8000 hours (LT80)^34,35^. Since the FQPO driving time does not exceed 30 seconds for one measurement, it is estimated that SpO_2_ monitoring is possible for about 2400 times or more, and is expected to increase further because of AC operation.

### Oxygen saturation level measurement with FQPO

The fiber-based OPD (FOPD) used a PEDOT:PSS electrode for its flexible and transparent properties. Supplementary Fig. 6 and Supplementary Table 2 compare the results of the OPD device structure which was used in this study and a transparent ITO electrode-based device with the same structure. It can be seen that the transmittance tendency of the PEDOT:PSS electrode, which is higher at shorter wavelengths and lower at longer wavelengths compared to ITO electrodes, follows the same trend as the external quantum efficiency (EQE) of each electrode (Supplementary Fig. 6b, c). In other words, the OPD with the PEDOT:PSS electrode, which is a flexible TCE, showed a similar level of light receiving efficiency as ITO. Based on the evaluation results of the device, in Fig. 1c, the FOPD was fabricated following the same process as the FQDLED.

In a photodiode, it is important to have responsivity characteristics at specific wavelengths. Figure 3a and Table 1 show the normalized EL intensity of the FQDLED and the responsivity of the FOPD. The red and green FQDLEDs showed peak wavelengths of 631 nm and 526 nm and a FWHM of 34 nm and 32 nm, and responsivity of 0.298 A/W and 0.291 A/W at each wavelength, respectively.

Another important photodiode factor is detectivity, which indicates the level at which signals can be measured against noise. This study used the detectivity on human skin (D^*^_human_) calculation method, employing the body temperature information summarized in a previous study^20^. It showed 7.01 × 10^10^ Jones and 6.84 × 10^10^ Jones at wavelengths of 630 nm and 530 nm, respectively. Those FOPD characteristics were similar to the OPD characteristics reported in previous wearable pulse oximetry reports, and therefore satisfied the favorable conditions for SpO_2_ monitoring regardless of body temperature.

Another important factor of reflection-type pulse oximetry is the angular characteristics, which affects the probability that light emitted at an angle in the lateral direction and incident on the photodiode is higher than that emitted from the front, because the light sources and photodiode are on the same plane. The FQDLED has the advantage of not only being thin and flexible but also having narrow FWHM characteristics, similar to that of an LED (Fig. 3a, b, Supplementary Fig. 6). Therefore, even when the angle is changed, it always has uniform spectral characteristics and lambertian characteristics. This means that the FQPO has superior wavelength selectivity (Fig. 3b, c).

**Fig. 4:**
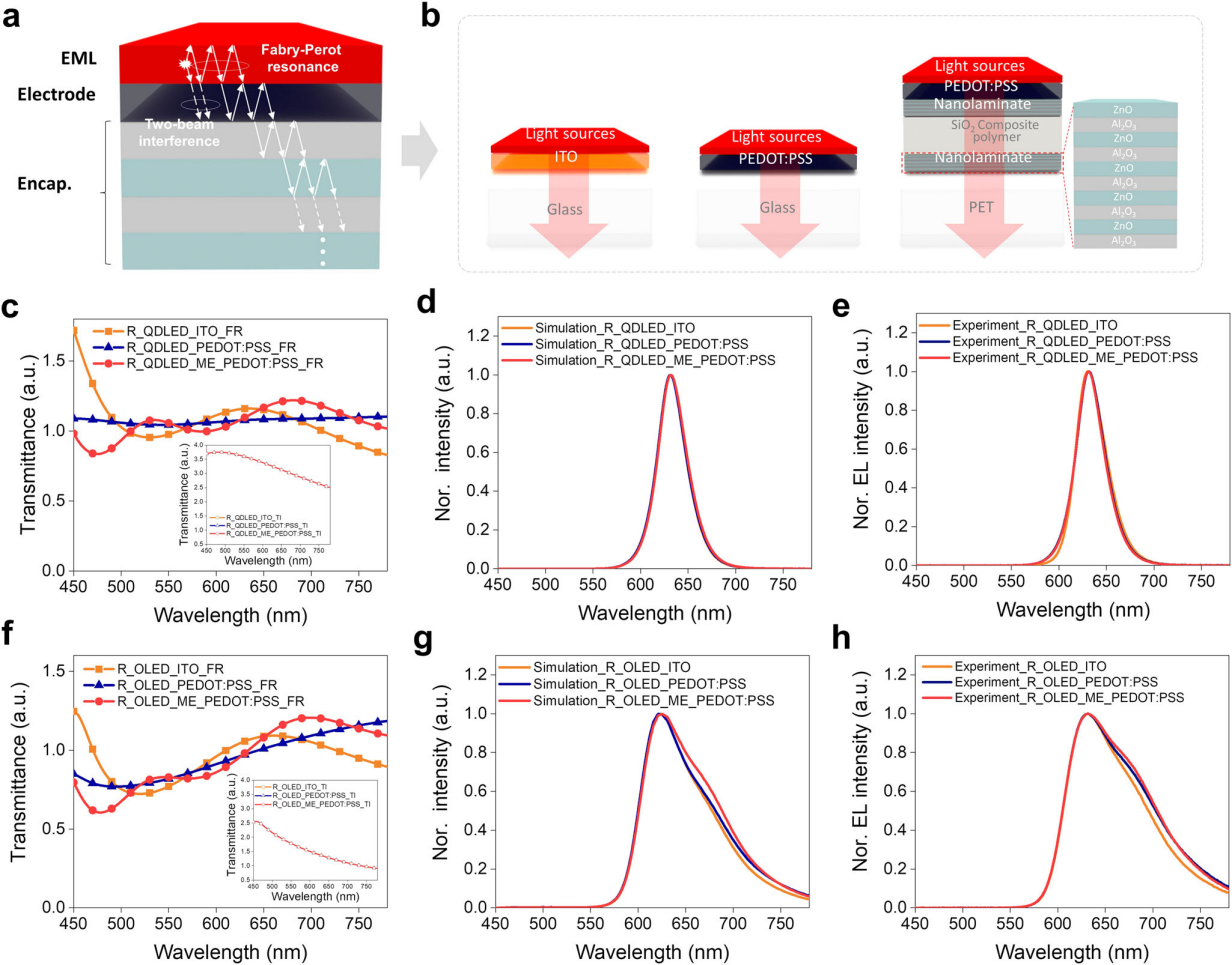
Wavelength selectivity according to the multilayer structure and the FWHM of the red light sources. **a** Schematic illustration and weak cavity phenomenon in the multilayer thin film structure. **b** Schematic illustration of the three different multilayer thin film structures used for the analysis of the light output characteristics according to the FWHM. **c**, **d**, **f**, **g** Simulation results for the FR and TI characteristics and spectral characteristics of the red QDLED and OLED light sources-based multilayer thin film structure. **e**, **h** Spectral characteristics based on the experiments of the QDLED and OLED light sources-based multilayer thin film structure.

**Fig. 5:**
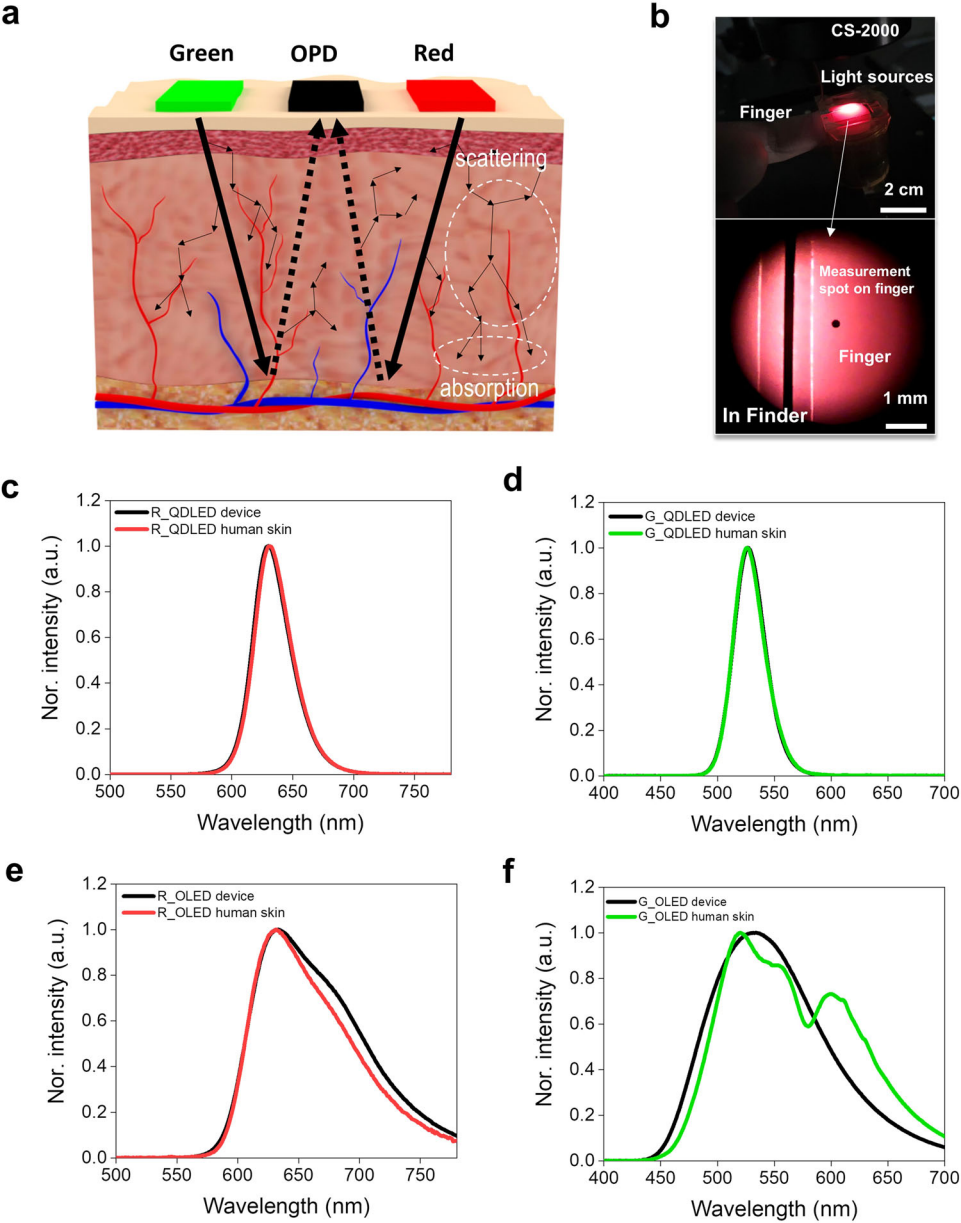
Wavelength selectivity of the reflected light from human skin according to the FWHM of the light sources. **a** Schematic illustration of the reflected light path based on the FQPO due to the scattering and absorption phenomena in human skin. **b** Photo of the experimental environment and method for measuring the reflected light characteristics from human skin. **c**–**f** Comparison of the spectral characteristics of the light emitted from the devices and the reflected light from the human skin according to the FWHM of the red and green QDLEDs and OLEDs.

Many previous reports have mainly focused on the flexible form factor of OLED light sources, but did not focus on the problem of broadening FWHM. The FQPO thereby has a distinct advantage because it can have high wavelength selectivity while retaining the advantages of the existing flexible form factor (Figs. 4 and 5, Supplementary Figs. 6 and 9, Table 1). Figure 3d shows a FQPO-based SpO_2_ monitoring device with uniform angular spectral characteristics. The monitoring system was configured as shown in Supplementary Fig. 8, referring to the method used in previous studies^20^. Here, the PPG signal measurement was conducted with a very small power consumption, of 0.03 mW and 0.031 mW for the red and green FQDLEDs, respectively (Fig. 3e). This shows that the FQPO can acquire vital signal information based on minimal noise, even with a very small radiant emittance of about 0.1 mW/cm^2^ or less, due to its lambertian characteristics (Fig. 3b, c).

As shown in Fig. 3d, for SpO_2_ monitoring, the FQPO-based PPG signal was obtained from the left index finger and the other PPG signal and SpO_2_ information were obtained from the right index finger using a commercial pulse oximetry device (CMS-50D plus, CONTEC MEDICAL SYSTEMS CO., LTD). To ensure stable vital signal information, measurements were performed while being driven with power consumptions of 0.69 mW and 0.91 mW for the red and green FQDLEDs, respectively, and 10 Hz and 30% duty cycle. Figure 3f shows that the PPG signal was very stable based on the left index finger and FQPO. In addition, the SpO_2_ level and bit per minute (BPM) were 99% and 80%, respectively. These results indicate that stable SpO_2_ monitoring was successful using the FQPO.

This study shows that practical SpO_2_ monitoring is possible, even using a thin fiber-based form factor with a very small amount of light power, from lambertian characteristics. Therefore, the narrow FWHM-based FQPO, which provides easy wavelength control through material size tuning, and has advantages of superb breathability, aesthetics and convenience using a fiber-based design, could be an optimized candidate for wearable pulse oximetry.

### Remarkable wavelength selectivity of multi-layer FQDLED structure on human skin with narrow FWHM

For SpO_2_ monitoring, specific light wavelength selectivity is very important. One of the peak wavelengths of the two light sources must have a relatively higher molar extinction coefficient of oxyhemoglobin than its deoxyhemoglobin counterpart, and the other light source must have the opposite tendency to achieve accurate SpO_2_ monitoring through a calibration process. In this section, we demonstrate there was superior wavelength selectivity for accurate SpO_2_ monitoring using the QD-based light sources on human skin.

To be wearable, it is essential to use flexible optoelectronic devices. Flexible TCE and multi-barrier encapsulation (ME) have been widely studied to ensure flexibility while preventing the penetration of water and oxygen^16,17,28^. That is, if EL occurs inside the device, the light is emitted through the multi-layer and is inevitably affected by the interference-based cavity effect.

Figure 4 shows the spectral characteristics of emitted light according to the FWHM of the light sources, for three different types of multi-layer structure. Here, $I_{{{{\mathrm{out}}}}}$ is the spectral characteristics of the out-coupled light from the device, $I_0$ is the emissive spectral characteristics in EML, and $G_{{{{\mathrm{cav}}}}}$ is the spectral characteristics of the cavity effect.

1 $$I_{{{{\mathrm{out}}}}}\left( \lambda \right) = I_0\left( \lambda \right) \times G_{{{{\mathrm{cav}}}}}\left( \lambda \right)$$

Figure 4a describes the optical cavity effect caused by the multi-layer, which can be divided into Fabry Perot resonance (FR) and two-beam interference (TI) phenomena. It can be summarized using Eqs. (2–5), where $f_{{{{\mathrm{FR}}}}}$ and $f_{{{{\mathrm{TI}}}}}$ are the FR and TI factor, $R_1$, $\phi _1$ are the reflectance and phase difference at the opposite interface of the emitted light direction, $T_2$, $R_2$, $\phi _2$ are the transmittance, reflectance, and phase difference at the boundary where the light is emitted, $\Delta \phi$ is the phase difference of the repeatedly reflected light at the upper and lower boundaries, *λ* is the wavelength of the light, $n_0$ and $d_0$ are the refractive index and thickness of the layer where the cavity occurs, and *θ* is the propagated light angle.

2 $$G_{{{{\mathrm{cav}}}}}\left( \lambda \right) = f_{{{{\mathrm{FR}}}}}\left( \lambda \right) \times f_{{{{\mathrm{TI}}}}}\left( {\lambda ;z} \right)$$

3 $$f_{{{{\mathrm{FR}}}}} = \frac{{T_2}}{{\left( {1 - \sqrt {R_1R_2} } \right)^2 + 4\sqrt {R_1} \sqrt {R_2} {{{\mathrm{sin}}}}^2\left( {\frac{{{{\Delta }}\phi }}{2}} \right)}}$$

4 $$f_{{{{\mathrm{TI}}}}} = 1 + R_1 + 2\sqrt {R_1} {{{\mathrm{cos}}}}\left( { + \phi _1 + \frac{{4\pi n_0z{{{\mathrm{cos}}}}\left( \theta \right)}}{\lambda }} \right)$$

5 $$\Delta \phi = + \phi _1 + \phi _2 + \frac{{2\pi }}{\lambda }\left( {2n_0d_0{{{\mathrm{cos}}}}\theta } \right)$$

This means that the light emitted from the EML is continuously affected by the refractive index and thickness of each layer, as well as the wavelength and propagation angle of the light before reaching the air, which will affect the spectral characteristics of the out-coupled light from the device.

In Fig. 4, QDLEDs and OLEDs with three different structures were used, a glass/ITO, glass/PEDOT:PSS, PET/ME/PEDOT:PSS (Fig. 4b), to compare the spectral characteristics of the out-coupled light where the light source has narrow and broad FWHM. Supplementary Fig. 9 shows the reflectance characteristics according to the composition of each layer. Even though the multi-layer was composed of transparent layers, a considerable amount of light was reflected from each layer, and thus a cavity phenomenon occurred. Figure 4c, f shows the results of the QDLED and OLED-based cavity effect simulation, which is dependent on the FR of each wavelength. Figure 4d, e, g, h are the normalized intensities of each wavelength for out-coupled light, based on simulation and experiment. It can be seen that the spectral characteristics of the out-coupled light of the QDLEDs with narrow FWHM in Fig. 4d, e are almost similar. However, the broad FWHM-based simulation and experiment results in Fig. 4g, h show a dissimilar tendency, which followed the FR tendency (Fig. 4f). These results indicate that narrow FWHM-based light can capture the specific wavelength well in the harsh environments of various kinds of multi-layer. In Supplementary Fig. 10, the green light source-based results were also similar to the red light source-based results, which adds to the credibility of the derived outcome.

Supplementary Fig. 7 shows the angular spectral characteristics under the same conditions as Fig. 4b. For the broad FWHM OLED light sources in Supplementary Fig. 7g–7l, the angular spectral characteristics continued to change depending on the conditions. However, as shown in Supplementary Fig. 7a–7f, the QDLED exhibited constant angular spectral characteristics. This indicates that a narrower FWHM-based light source has better ability to capture a specific light wavelength, regardless of the angle. Using light sources with specific wavelength to the high molar extinction coefficient of oxyhemoglobin or deoxyhemoglobin determines the accuracy of SpO_2_ monitoring. The QDLED optoelectronic device can be optimized for a wearable pulse oximeter.

Figure 5 shows analysis results for the wavelength selectivity of human skin. Human skin consists of epidermis, dermis, lipids, and muscle layers, and inside these are complex tissues and structures such as mitochondria, collagen fiber bundles, lysosomes, and the lipid-water interface. When reflection-type pulse oximeter is applied, the light scattering and absorption phenomena caused by these features is included in the OPD. Also, according to previous reports, these phenomena tend to decrease with longer wavelengths^36^. That is, the wavelength dependent phenomena may result in different spectral information from the light source entering the OPD.

In this study, a simple experiment was used to analyze the actual spectral information passing through the human skin tissue and then entering the FOPD (Fig. 5c). A FQDLED and FOLED were fabricated and used for the evaluation. The out-coupled light from the finger surface was measured through a spectrometer (Konica Minolta Inc. CS-2000), and measured again after the out-coupled light from the light source was incident on the surface of the human index finger. For the narrow FWHM-based FQDLED, the spectral information of out-coupled light from the device and the human skin were almost the same, in spite of the scattering and absorption phenomenon inside the skin, as shown in Fig. 5c, d. In contrast, for the broad FWHM-based FOLED, the spectral information of the out-coupled light from the device and the human skin were significantly different. This was due to the different scattering and absorption phenomena of each wavelength through the complex tissues in the skin. Notably, the spectral modification observed in the green area was similar to the previously reported results of absorption coefficient^37^. Such spectral modification can have a very sensitive effect on the performance of a wearable pulse oximeter.

The formula for SpO_2_ level and modulation ratio ($R_{{{{\mathrm{os}}}}}$) has been reported in previous studies based on the photon diffusion theory^38^, and are provided in Eqs. (6), (7), where $\varepsilon _{{{{\mathrm{Hb}}}}}$ and $\varepsilon _{{{{\mathrm{HbO}}}}2}$ are the molar extinction coefficient of the deoxyhemoglobin and oxyhemoglobin, $\lambda _1$ and $\lambda _2$ are the wavelength of the light in pulse oximetry, *c* is a variable for reduced scattering coefficient and absorption in human skin, and ${{{\mathrm{AC}}}}_1$, ${{{\mathrm{AC}}}}_2$, ${{{\mathrm{DC}}}}_1$, ${{{\mathrm{DC}}}}_2$ are alternate current (AC) and direct current (DC) information in the PPG signal measured for each light.

6 $${{{\mathrm{SpO}}}}_2\left( {R_{{{{\mathrm{os}}}}}} \right) = \frac{{\varepsilon _{{{{\mathrm{Hb}}}}}\left( {\lambda _1} \right) \cdot c - \varepsilon _{{{{\mathrm{Hb}}}}}\left( {\lambda _2} \right) \cdot R_{{{{\mathrm{os}}}}}}}{{\left( {\left( {\varepsilon _{{{{\mathrm{Hb}}}}}\left( {\lambda _1} \right) - \varepsilon _{{{{\mathrm{HbO}}}}2}\left( {\lambda _1} \right)} \right)} \right. \cdot c + \left( {\left( {\varepsilon _{{{{\mathrm{HbO}}}}2}} \right.\left( {\lambda _2} \right) - \varepsilon _{{{{\mathrm{Hb}}}}}\left( {\lambda _2} \right)} \right) \cdot c}}$$

7 $$R_{os} = \frac{{{{{\mathrm{AC}}}}_1/{{{\mathrm{DC}}}}_1}}{{{{{\mathrm{AC}}}}_2/{{{\mathrm{DC}}}}_2}}$$

In the $R_{{{{\mathrm{os}}}}}$-based calibration process, the linearity and slope of the calibration curve reflect the accuracy and resolution of the pulse oximetry^36^. Here, the denominator and numerator of $R_{{{{\mathrm{os}}}}}$ contain the oxyhemoglobin and deoxyhemoglobin information, respectively. Using a broad FWHM-based light source blurs the boundaries of the oxyhemoglobin and deoxyhemoglobin information, and the slope and linearity of the $R_{{{{\mathrm{os}}}}}$-based calibration curve can change according to the SpO_2_ level. In contrast, narrow FWHM-based pulse oximetry results in a uniform $R_{{{{\mathrm{os}}}}}$-based calibration curve with superior wavelength selectivity, due to the narrow and uniform spectral characteristics. As a result, it provides the high accuracy and resolution required for wearable pulse oximetry, as shown in Figs. 4, 5.

As the wearable market expands, interest in thin and flexible wearable pulse oximetry is increasing day by day. Based on its narrow FWHM characteristics, QDLED-based wearable pulse oximetry can enable accurate and high resolution SpO_2_ monitoring, as well as a thin and flexible platform.

### Low DC noise signal waveguide mode with fiber substrate

**Fig. 6:**
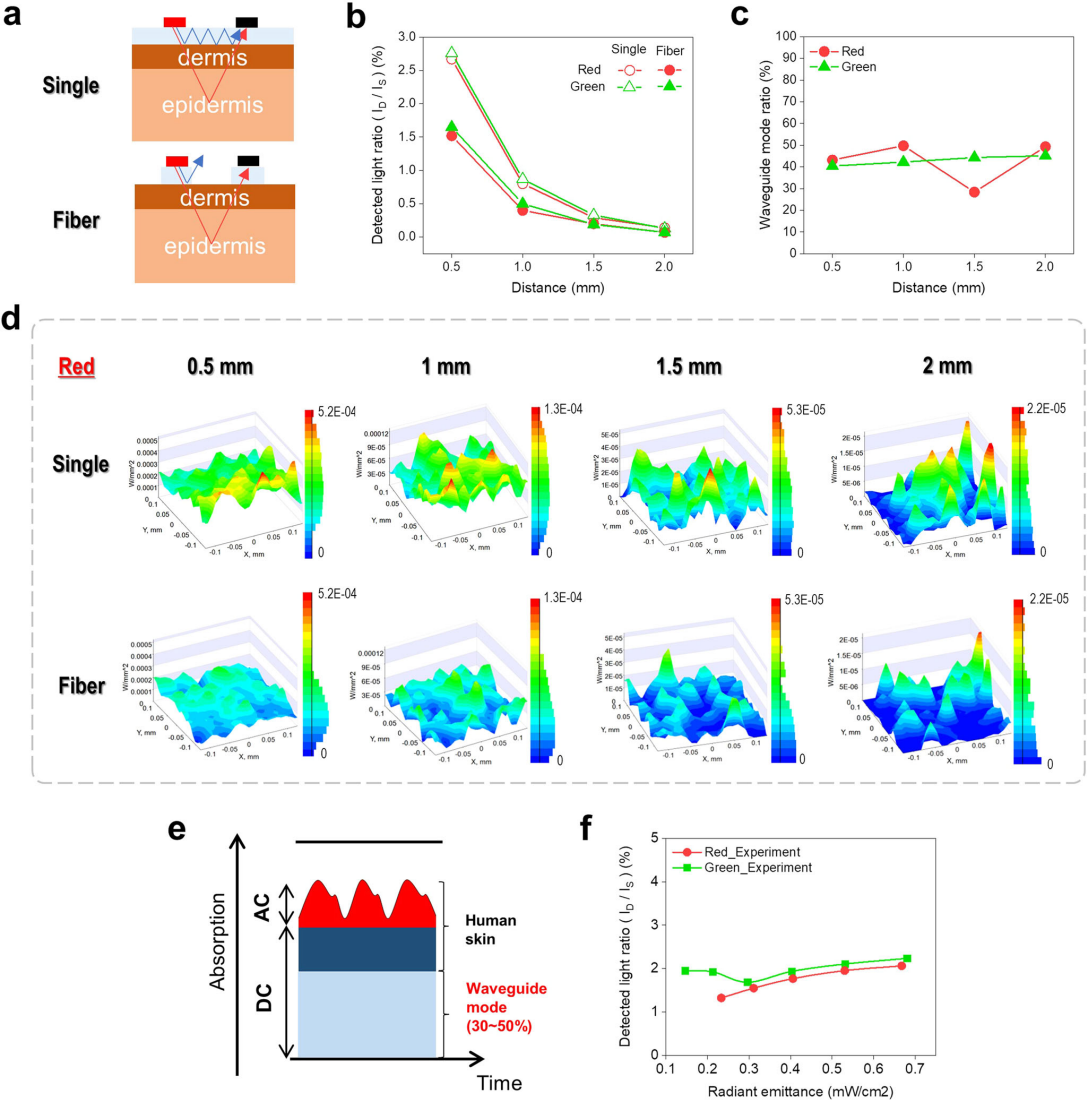
Superior PPG sensitivity based on fiber substrate. **(a)** Schematic illustration of the waveguide mode optical path in single and fiber substrates based on reflection-type pulse oximetry. **(b)** Comparison of the detected light ratio by distance of the red and green light sources in the single and fiber substrates. **(c)** Waveguide mode ratio of the red and green light sources by distance. **(d)** Comparison of the radiance by distance in single and fiber substrates of the red light sources (Area: 0.2 ×0.2 mm^2^). **(e)** Absorption diagram of the PPG signal and waveguide mode ratio in DC signal. **(f)** Experimental result showing the detected light ratio by radiant emittance.

The refractive index of conventional polymer-based flexible substrates such as PET ($n_{{{{\mathrm{PET}}}}}$ = 1.57) is greater than the refractive index of air ($n_{{{{\mathrm{air}}}}}$ = 1.57) as well as the refractive index of the epidermis of human skin ($n_{{{{\mathrm{epidermis}}}}}$ = 1.57). In other words, a waveguide mode due to total reflection exists inside the substrate, and it can appear as a noise signal because it is included in the DC component of the PPG signal in reflection-type pulse oximetry. The influence of the waveguide mode in PPG signal was analyzed through simulation and experiment for a 2D single substrate type and a 1D fiber substrate type in a reflection-type pulse oximetry, as shown in Fig. 6. Fig. 6A shows the optical path of the waveguide mode light source in the single substrate and fiber substrate. Using a ray-tracing-based simulation, the analysis considered the PET, dermis, and epidermis layers under a light source and detector, as shown in Supplementary Fig. 11a. It was assumed that there was no lipid, muscle, or bone layer, referring to the results of previous studies that indicated their scattering influence was relatively small^39^. Both the single substrate and fiber substrate were subjected to 100,000 light rays ($I_{{{\mathrm{S}}}}$) output from the light source, and the amount of light ($I_{{{\mathrm{D}}}}$) incident into the detector was measured at different distances (*d*) of 0.5 mm, 1 mm, 1.5 mm, and 2 mm (Supplementary Fig. 11a).

Table 2 and Fig. 6b are the detected light ratios ($I_{{{\mathrm{D}}}}/I_{{{\mathrm{S}}}}$) expressed as the ratio of $I_{{{\mathrm{D}}}}$ and $I_{{{\mathrm{S}}}}$, and Fig. 6d is the result for the red light source-based simulation. The results show that the detected light ratio could be clearly distinguished according to the substrate, rather than the wavelength, and that the number of rays extracted from the single substrate was much larger. It can be predicted that most of these differences are DC noise signals due to the waveguide mode in the single substrate, and if the difference is approximately expressed as a waveguide mode ratio, it can be summarized by Eq. (8).

8 $${{{\mathrm{Waveguide}}}}\;{{{\mathrm{mode}}}}\;{{{\mathrm{ratio}}}} = \left( {\frac{{I_{{{\mathrm{D}}}}\;{{{\mathrm{on}}}}\;{{{\mathrm{a}}}}\;{{{\mathrm{single}}}}\;{{{\mathrm{sub}}}}. - I_{{{\mathrm{D}}}}\;{{{\mathrm{on}}}}\;{{{\mathrm{a}}}}\;{{{\mathrm{fiber}}}}\;{{{\mathrm{sub}}}}.}}{{I_{{{\mathrm{D}}}}\;{{{\mathrm{on}}}}\;{{{\mathrm{a}}}}\;{{{\mathrm{single}}}}\;{{{\mathrm{sub}}}}.}}} \right) \times 100$$

**Table 2:** Results of the detected light ratio simulation.

		Detected light ratio (*I*_D_ / *I*_S_) (%)			
		0.5 mm	1 mm	1.5 mm	2 mm
Single	Red	2.67	0.80	0.29	0.14
	Green	2.76	0.87	0.33	0.13
Fiber	Red	1.52	0.40	0.20	0.07
	Green	1.65	0.50	0.19	0.07

Figure 6c shows the waveguide mode ratio, which was about 30% to 50% and exhibited little change with distance. That is, in a single substrate-based reflection-type pulse oximetry, nearly half of the light was from the waveguide mode. This is a significant proportion, considering that the proportion of AC signals in the PPG signal we are actually interested in, information indicating the volume change in blood vessels, is very small, as shown in Fig. 6e.

When R_OS_ is obtained for SpO_2_ measurement, it is divided into the DC signals of the two lights, to reduce the influence of light intensity. Therefore, unlike the wavelength-dependent reflected light from human skin, the light in the waveguide mode has no wavelength-dependent tendency, which will be a noise factor in the calibration process (Fig. 6c). In other words, the fiber substrate-based reflection-type pulse oximetry can provide superior SpO_2_ measurement characteristics because it has no noise signal from the waveguide mode.

Figure 6f provides the value that was obtained by experimentally measuring the FQDLED-based detected ratio, to verify the corresponding simulation result. In the measurement method, the light reflected from the index finger was measured at a distance of about 0.7 mm as shown in Fig. 5b. This indicates the detected light ratio was slightly higher than the simulation result of the device in Fig. 6b. The reason for the slight error is that inside the human skin, there is some additional scattering light from fat, muscle and bone, as well as the dermis and epidermis^39^. Based on these results, the simulation result is quite reliable, and when implementing a fiber substrate-based reflection-type pulse oximetry, the DC noise signal introduced by the waveguide mode has little effect on the PPG quality.

In summary, we implemented FQPO, which is suitable for mass production and PPG signal measurement, with a low power consumption (about 0.03 mW) for SpO_2_ monitoring using lambertian characteristics. We also demonstrated through experiments and simulations that the narrow FWHM (about 30 nm) light source-based uniform spectral properties are important in terms of wavelength selectivity and accurate SpO_2_ measurement in harsh environments, where the light source has to pass through complex multi-layer thin film structures and human skin. In addition, the analysis and interpretation of a ray tracing simulation confirmed that the fiber-based pulse oximetry is capable of obtaining a PPG signal with waveguide mode-free characteristics for accurate SpO_2_ measurement (Table 3).

**Table 3:** Comparison of key performance results of the FQPO in this study and previously reported wearable pulse oximetry.

Platform	Electrode	Light sources	Peak $\lambda$ (nm)	FWHM (nm)	SpO_2_	Purpose	Year, Ref.
Fiber	PEDOT:PSS	red, green QDLED	631/526	34/32	O	fiber, ${{{{\lambda }}}}$ selectivity, sensitivity	Ours 2023
Glass	ITO	red, green OLED	626/532	>80	O	all organic	2014^40^
Patch	ITO	red, green PLED	609/517	>50	O	ultra thin	2016^19^
Patch	Grephene	red, green QDLED	618/520	38/35	–	stretchable, QD	2017^41^
Patch	ITO	red, green PLED	611/520	>70	O	blade coating	2017^42^
Patch	ITO	red, NIR OLED	612/725	>80	O	mapping, high accuracy	2018^10^
Patch	IZO	red, green OLED	620/510	>70	O	low power consumption	2018^20^
Patch	PEDOT:PSS	color filter & ambient light	610/525/740	>60	O	ambient light, color filter	2020^21^
Patch	ITO	yellow PLED	590	>100	–	self-power	2021^22^
Patch	IZO	green OLED	530–540	–	–	stretchable, motion artifacts rejection	2022^43^

The modern medical paradigm is gradually moving from hospital-based treatment to real-time monitoring-based prevention. Wearable pulse oximetry is a very attractive medical device for this application. However, the successful implementation of a thin and flexible platform base will be insufficient if the measurement accuracy is lower than that of a conventional device. The high wavelength selectivity and waveguide mode-free characteristics of the FQPO presented in this study indicate it can play a very important role in real-time health monitoring as a wearable opto-electronic device. It is hoped that the main ideas and directions presented here will contribute to the development of wearable pulse oximetry research, and increase the fundamental measurement accuracy when using light sources for SpO_2_ monitoring, as well as flexible form-factor devices.

## Methods

### Solution preparation

PEDOT:PSS (Clevios PH1000), ZnO NPs, and PEI were prepared by a method similar to previous studies^24–26^. The PEDOT:PSS solution was doped with 5 wt% dimethyl sulfoxide (DMSO) and 0.5 wt% Zonyl FS-300 in order to improve conductivity and hydrophobic interface coating. ZnO NPs utilized the following synthesis method. First, prepared 1.51 g of potassium hydroxide (KOH) and 3 g of zinc acetate dihydrate (Zn(Ac)_2_ ∙ 2H_2_O) were dissolved in 60 mL and 120 mL methanol (MeOH) solutions, respectively. Afterwards, for the mixture reaction, heat was applied at 65 °C and the KOH solution was dropped and stirred into the (Zn(Ac)_2_ ∙ 2H_2_O) solution in a 3-neck round flask for 140 min. Then, a precipitate of ZnO NPs was obtained by centrifugation at 4000 rpm for 30 min. Finally, the ZnO NPs solution was obtained by dissolving in 1-butanol at 25 mg/mL.

The PEI was prepared by dissolving in 0.06 wt% in 2-methoxyethanol. The QD solution was purchased from Global ZEUS, and dissolved in a solution of toluene at 100 mg/mL, and exhibited photoluminescence characteristics of about 630 nm and 530 nm peak wavelength with a cadmium selenide/zinc sulfide (CdSe/ZnS) structure. After that, it was diluted with 10 mg/mL for use in the process. As the OPD active layer, PBDBT-2F, which is the donor role, and IT-4F, which is the acceptor role, were diluted with a concentration of 20 mg/mL in chlorobenzene:1-8-diiodoctane solution with a volume ratio of 99.5:0.5 at a 1:1 ratio.

### Transferable encapsulation fabrication

First, we prepared a 125 nm-thickness PET film for device fabrication and a 20 mm adhesive PET film (SOOKWANG TTI Co. Ltd) to enable transferable encapsulation. Then, the PET films were used for an atomic layer deposition (ALD) process to fabricate 30 nm inorganic/organic nanolaminate-based thin films with Al_2_O_3_/ZnO (3 nm/3 nm) at 70 °C heating in the chamber. Thereafter, the SiO_2_ composite polymer was coated with a 425 nm thickness by spin-coating for 3 s at 5000 rpm with acceleration 30 s, followed by annealing for 10 min at 70 °C in the ALD chamber. Then, the 30 nm-thickness nanolaminate-based thin film (Al_2_O_3_/ZnO, 3 nm/3 nm) process was repeated to form a 1.5 dyads ME film. Finally, fabrication of the QDLED and OPD was performed on the processed 125 μm-thickness PET film, and then, the processed 20 μm-thickness adhesive PET film was attached to the upper part of the processed 125 μm-thickness PET film through a roll-to-roll process.

### FQDLED fabrication

A 135 nm-thickness PEDOT:PSS electrode was processed at room temperature in air environment on the ME thin film processed 125 μm-thickness PET film by the spin-coating method, which was conducted at 1000 rpm for 40 s with 5 s acceleration, then annealed at 140 °C for 30 min. Next, a 20 nm-thickness ZnO NPs thin film was formed by spin-coating at 2000 rpm, acceleration 0.5 s for 40 s, and an annealing process at 140 °C for 30 min. After that, the PEI solution was spin-coated at 5000 rpm, acceleration 5 s, and 40 s, and annealed at 140 °C for 5 min, and the ethanol (EtOH) rinse used in previous studies was omitted, to form a thick insulating layer^24–26^. Then, the QD solution was spin-coated at 3000 rpm, acceleration 1 s for 40 s, and annealed 140 °C for 30 min to form a 20 nm-thickness QD layer. Subsequently, 4,4′,4″-tris (N-carbazolyl) triphenylamine (TCTA) (40 nm)/molybdenum oxide (MoO_3_) (10 nm)/aluminum (Al, 100 nm) was formed through thermal evaporation vacuum (5 × 10^−6^ torr) deposition. Finally, after performing the transferable encapsulation process, the FQDLED was completed using a thin and very sharp scissors-based slitting process.

### FOPD fabrication

The process was carried out similarly to the FQDLED. First, a PEDOT:PSS layer was formed in the same way as the FQDLED. After that, a 20 nm-thickness ZnO NPs layer was spin-coated at 2000 rpm, acceleration 0.5 s for 40 s and annealed on a hot plate at 100 °C for 15 min. After that, a PBDBT-2F:IT-4F solution was spin-coated at 1500 rpm, acceleration 5 s for 60 s as the OPD active layer, and annealed on a hot plate at 100 °C for 15 min. Next, MoO_2_ (10 nm)/silver (Ag, 100 nm) was deposited through a thermal evaporation process. Finally, FOPD was completed using the same transferable encapsulation process and slitting process as the FQDLED.

### Device characteristics and SpO_2_ monitoring

The current density–voltage–luminance(J–V–L) characteristics of the optoelectronic devices were measured by source measurement unit (Keithley 2400) and spectro-radiometer (CS2000, Konica Minolta Inc.). The J–V characteristics of the OPD were obtained with a source measure unit (Keithley 238) and a photovoltaics spectral response measurement system (CEP-25ML, Bunkoukeiki) in the DC mode. A bending machine (Scientetown Inc.) was used to evaluate flexibility through repeated bending. The sheet resistance of the electrode was measured with a four-point probe system (Keithley 2750, USA). A spectrophotometer (UV-2550, Shimadzu, Japan) was used to measure optical transmittance and reflectivity. The OLED lifetime test system (Polaronix M6000, McScience, Korea) was used to measure the lifespan of the FQDLEDs. PCB was used to measure the PPG signal after design and purchase through BoardLab. A commercial pulse oximetry device, CMS-50D + , was purchased from CONTEC MEDICAL SYSTEM Co., LTD and used for real-time SpO_2_ level and BPM monitoring.

### Optical characteristics of the reflected light from human skin

The spectral characteristics measurements were performed using the source measurement unit (Keithley 2400) and spectro-radiometer (CS2000, Konica Minolta Inc.) in the same way as the J-V-L characteristics measurement. Customized substrates and hollow glass were prepared for measurement, and the manufactured FQDLED and FOLED were attached. To measure the light reflected from the human skin, the light emission direction was directed in the opposite direction of the spectro-radiometer. After that, the index finger was closely attached to the device, and the measurement aperture of the spectro-radiometer was arranged on the index finger surface to measure the spectral characteristics of the out-coupled light vertically, which is scattered and absorbed inside the human skin.

### Optical simulation

Weak cavity simulation was calculated using MATLAB (MathWorks). Both The FR factor and TI factor were obtained from calculation through simulation. The refractive index of each wavelength of each material was experimentally obtained through a spectroscopic ellipsometer (M2000D) for use in the simulation. The simulation was only calculated in the normal direction of the device, and it was assumed that there was no effect due to the difference between the PET substrate and the glass substrate.

LightTools were used for the ray-tracing-based geometrical optics simulation. PET, dermis, epidermis, and light source information used the embedded information in the simulation tool. A 125 μm-thick PET film was used, the same as in the experimental conditions, and 270 μm-thick and 680 μm-thick epidermis and dermis were applied, respectively, referring to the results of previous studies. For the comparative simulation, the PET was assumed to be a single substrate and a fiber substrate, and a surface light source was located on the top of the PET. To obtain the number of rays detected by distance, a detector was located at distances of 0.5 mm, 1 mm, 1.5 mm, and 2 mm, respectively.

## Supplementary information

Fiber-based Quantum-dot Pulse Oximetry for Wearable Health Monitoring with High Wavelength Selectivity and Photoplethysmogram Sensitivity

## Data Availability

The data that support the findings of this study are available from the corresponding author upon reasonable request.

## References

[1] Song J, Lee H, Jeong EG, Choi KC, Yoo S (2020). Organic light-emitting diodes: pushing toward the limits and beyond. Adv. Mater..

[2] Kim CH (2021). OLED opportunity in healthcare with the pulse oximeter. Inf. Disp..

[3] Jeon Y, Lee D, Yoo H (2022). Recent advances in metal-oxide thin-film transistors: flexible/stretchable devices, integrated circuits, biosensors, and neuromorphic applications. Coatings.

[4] Gepner Y (2022). Utilizing wearable sensors for continuous and highly-sensitive monitoring of reactions to the BNT162b2 mRNA COVID-19 vaccine. Commun. Med..

[5] Berwal D, Kuruba A, Shaikh AM, Udupa A, Baghini MS (2022). SpO_2_ measurement: non-idealities and ways to improve estimation accuracy in wearable pulse. IEEE Sens..

[6] Mirjalali S, Peng S, Fang Z, Wang CH, Wu S (2022). Wearable sensors for remote health monitoring: potential applications for early diagnosis of covid-19. Adv. Mater. Technol..

[7] Nitzan M, Romem A, Koppel R (2014). Pulse oximetry: fundamentals and technology update. Med. Devices: Evid. Res..

[8] Mendelson Y (1992). Pulse oximetry: theory and applications for noninvasive monitoring. Clin. Chem..

[9] Chan ED, Chan MM, Chan MM (2013). Pulse oximetry: Understanding its basic principles facilitates appreciation of its limitations. Respir. Med..

[10] Khan Y (2018). A flexible organic reflectance oximeter array. Proc. Natl Acad. Sci. USA.

[11] Choi S (2017). Highly flexible and efficient fabric-based organic light-emitting devices for clothing-shaped wearable displays. Sci. Rep..

[12] Park Y (2022). Cell proliferation effect of deep-penetrating microcavity tandem NIR OLEDs with therapeutic trend analysis. Sci. Rep..

[13] Jeon Y (2019). Sandwich-structure transferable free-form OLEDs for wearable and disposable skin wound photomedicine. Light. Sci. Appl..

[14] Triana MA, Restrepo AA, Lanzafame RJ, Palomaki P, Dong Y (2020). Quantum dot light-emitting diodes as light sources in photomedicine: photodynamic therapy and photobiomodulation. J. Phys. Mat..

[15] Choi MK, Yang J, Hyeon T, Kim D-H (2018). Flexible quantum dot light-emitting diodes for next-generation displays. npj Flex. Electron..

[16] Jeong SY (2021). Foldable and washable textile-based OLEDs with a multi-functional near-room-temperature encapsulation layer for smart e-textiles. npj Flex. Electron..

[17] Jeong EG, Jeon Y, Cho SH, Choi KC (2019). Textile-based washable polymer solar cells for optoelectronic modules: toward self-powered smart clothing. Energy Environ. Sci..

[18] Kwon JH (2019). Design of highly water resistant, impermeable, and flexible thin-film encapsulation based on inorganic/organic hybrid layers. ACS Appl. Mater. Interfaces.

[19] Yokota T (2016). Ultraflexible organic photonic skin. Sci. Adv..

[20] Lee H (2018). Toward all-day wearable health monitoring: an ultralow-power, reflective organic pulse oximetry sensing patch. Sci. Adv..

[21] Han D (2020). Pulse oximetry using organic optoelectronics under ambient light. Adv. Mater. Technol..

[22] Jinno H (2021). Self-powered ultraflexible photonic skin for continuous bio-signal detection via air-operation-stable polymer light-emitting diodes. Nat. Commun..

[23] Song YJ (2020). Fibertronic organic light-emitting diodes toward fully addressable, environmentally robust, wearable displays. ACS Nano.

[24] Kwon S (2018). Weavable and highly efficient organic light-emitting fibers for wearable electronics: a scalable, low-temperature process. Nano Lett..

[25] Hwang YH (2021). Bright-multicolor, highly efficient, and addressable phosphorescent organic light-emitting fibers: toward wearable textile information displays. Adv. Funct. Mater..

[26] Hwang YH (2022). High-performance and reliable white organic light-emitting fibers for truly wearable textile displays. Adv. Sci..

[27] Kwon S (2015). High luminance fiber-based polymer light-emitting devices by a dip-coating method. Adv. Electron. Mater..

[28] Jeong EG, Han YC, Im HG, Bae BS, Choi KC (2016). Highly reliable hybrid nano-stratified moisture barrier for encapsulating flexible OLEDs. Org. Electron..

[29] Ko K, Lee HB, Kang J (2020). Flexible, wearable organic light‐emitting fibers based on PEDOT:PSS/Ag‐fiber embedded hybrid electrodes for large‐area textile lighting. Adv. Mater. Technol..

[30] Kim K (2019). A study on dispersion behaviors of fume particles in laser cutting process of optical plastic thin films. J. Semiconductor Disp. Technol..

[31] Wang R (2022). Unravelling the bending stability of flexible quantum-dot light-emitting diodes. Flex. Print. Electron..

[32] Shen P, Li X, Cao F, Ding X, Yang X (2018). Highly efficient, all-solution-processed, flexible white quantum dot light-emitting diodes. J. Mater. Chem. C.

[33] Son DI, Kim HH, Hwang DK, Kwon S, Choi WK (2014). Inverted CdSe–ZnS quantum dots light-emitting diode using low-work function organic material polyethylenimine ethoxylated. J. Mater. Chem. C.

[34] Li Y (2021). Non-fullerene acceptor organic photovoltaics with intrinsic operational lifetimes over 30 years. Nat. Commun..

[35] Du X (2019). Efficient polymer solar cells based on non-fullerene acceptors with potential device lifetime approaching 10 years. Joule.

[36] Kaniusas, E. *Biomedical Signals and Sensors II: Linking Acoustic and Optic Biosignals and Biomedical Sensors*. (Springer, 2015).

[37] Lister T, Wright PA, Chappell PH (2012). Optical properties of human skin. J. Biomed. Opt..

[38] Ruíz AAB (1991). Simple photon diffusion analysis of the effects of multiple scattering on pulse oximetry. IEEE Trans. Biomedi. Eng..

[39] Chatterjee S, Kyriacou PA (2019). Monte carlo analysis of optical interactions in reflectance and transmittance finger photoplethysmography. Sensors.

[40] Lochner CM, Khan Y, Pierre A, Arias AC (2014). All-organic optoelectronic sensor for pulse oximetry. Nat. Commun..

[41] Kim TH (2017). Fully stretchable optoelectronic sensors based on colloidal quantum dots for sensing photoplethysmographic signals. ACS Nano.

[42] Han D (2017). Flexible blade-coated multicolor polymer light-emitting diodes for optoelectronic sensors. Adv. Mater..

[43] Lee GH (2022). Stretchable PPG sensor with light polarization for physical activity – permissible monitoring. Sci. Adv..

