# Supplementary material for: Fiber-based quantum-dot pulse oximetry for wearable health monitoring with high wavelength selectivity and photoplethysmogram sensitivity
Source: Npj Flex Electron. 2023 Mar 17;7(1):15. doi: 10.1038/s41528-023-00248-1 (PMC10020774; doi:10.1038/s41528-023-00248-1)
Supplement: Supplementary file 1 — Fiber-based Quantum-dot Pulse Oximetry for Wearable Health Monitoring with High Wavelength Selectivity and Photoplethysmogram Sensitivity [file 41528_2023_248_MOESM1_ESM.pdf]

Supplementary Information

Fiber-based Quantum-dot Pulse Oximetry for Wearable Health Monitoring with High Wavelength Selectivity and Photoplethysmogram Sensitivity

Ho Seung Lee[1], Byeongju Noh[1], Seong Uk Kong[1], Yong Ha Hwang[1], Ha-Eun Cho[1], Yongmin Jeon[2] and Kyung Cheol Choi[1*]*

[1]School of Electrical Engineering, Korea Advanced Institute of Science and Technology (KAIST), Daejeon 34141, Republic of Korea.

[2]Department of Biomedical Engineering, Gachon University, Seongnam 13120, Republic of Korea.

Email: kyungcc@kaist.ac.kr (Kyung Cheol Choi), yongmin@gachon.ac.kr (Yongmin Jeon)

Supplementary Methods

Strain calculation method according to bending radius.

The strain according to bending radius was calculated using Supplementary Equation (1), where R is the bending radius, and *d*/2 is the neutral axis to device thickness information.

$$\text{Strain}\ (\varepsilon, \%) = \frac{d}{2} \times \frac{1}{R} \qquad (1)$$

In Supplementary Figure 3c, 3d, since PEDOT:PSS is coated on 125 μm-thickness PET film, the neutral axis to device thickness is 62.5 μm and the bending radii are 10 mm, 2.5 mm, 1.5 mm. The resulting strains calculated with Supplementary Equation (1) are about 0.63 %, 2.5 % and 4.17 %, respectively.

When the transferable encapsulation process was performed as in Supplementary Figure 3e, the total PET thickness was 145 μm, and it can be seen that the device located in the middle is close to the neutral axis, within 20 μm (72.5-20 μm). Accordingly, when the bending radius was 10 mm, 6 mm, 5 mm and 4 mm, the strain was about 0.5 %, 0.9 %, 1.0 % and 1.3 %, respectively, and it can be said that it was as flexible as the distance from the neutral axis to the device. It was less flexible than the PEDOT:PSS electrode, because of the inorganic film deposited for encapsulation. In other words, the FQDLED prepared with the transferable encapsulation process has more flexible characteristics.

Supplementary Figures

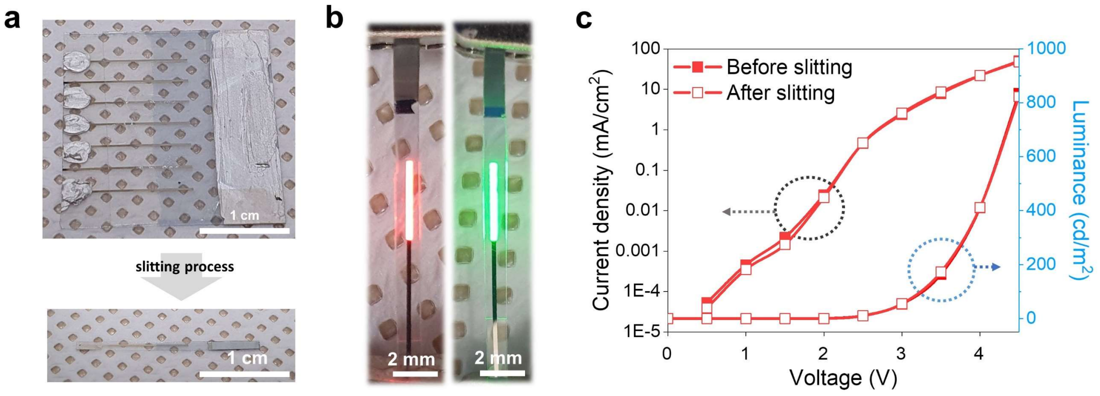

Supplementary Figure 1. Characteristics of the FQDLED according to the slitting process. (a) Photos before and after slitting the FQDLED. **(b)** Photo of the red and green FQDLEDs after slitting. **(c)** Comparison of current density-voltage (J-V) and luminance-voltage (L-V) characteristics before and after the slitting process.

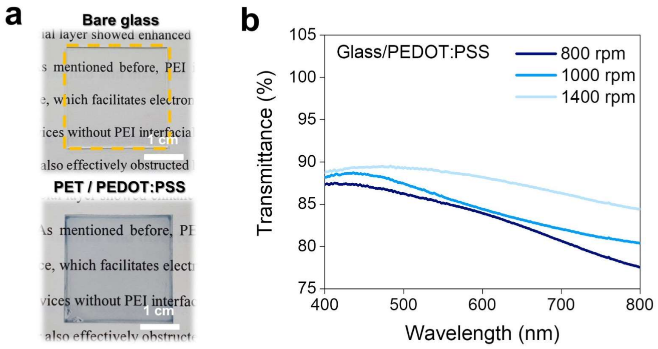

Supplementary Figure 2. Transmittance and sheet resistance characteristics of the PEDOT:PSS electrode. (a) Photo of transparent PEDOT:PSS electrode compared with bare glass. **(b)** Transmittance characteristics of the PEDOT:PSS according to spin-coating rpm. **(c)** Sheet resistance characteristics and thickness of the PEDOT:PSS according to spin-coating rpm.

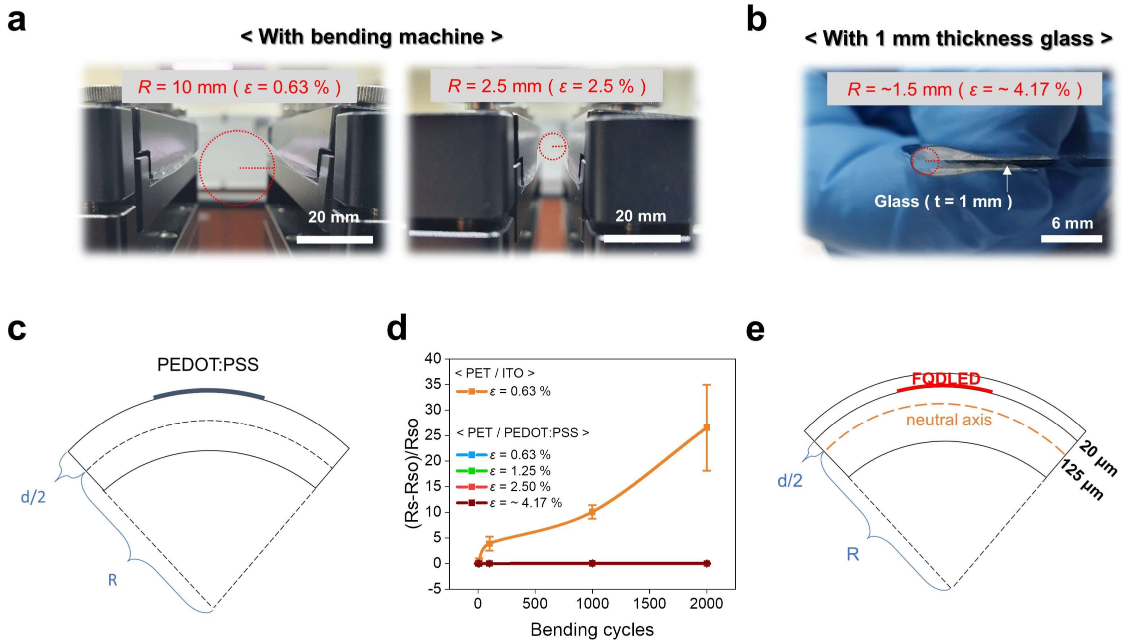

Supplementary Figure 3. Flexibility test based on bending machine. (a) Bending test photos with bending machine (strain = 0.63 %, 2.5 %). **(b)** Bending test photo using 1 mm thick glass (strain is about 4.17 %). **(c)** Schematic illustration of the strain calculation for the PEDOT:PSS electrode. **(d)** Rate of change in resistance by strain and bending cycle of the ITO and PEDOT:PSS electrode (PET thickness = 125 μm, maximum number of bending cycles is 2000 times). **(e)** Schematic illustration of strain calculation of FQDLED.

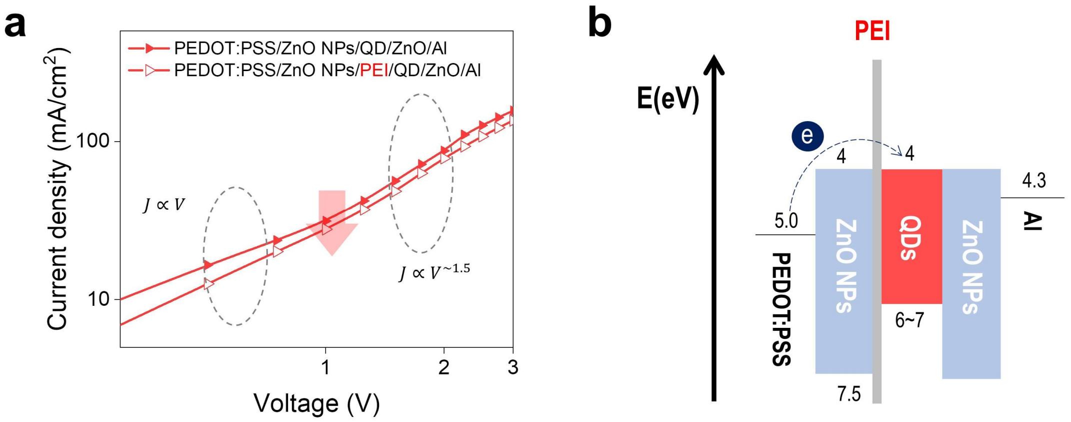

Supplementary Figure 4. Electron only device characteristics according to the PEI insulating layer. (a) Current density-Voltage (J-V) characteristics. **(b)** Schematic energy diagram of the PEI insulating layer-based electron only device.

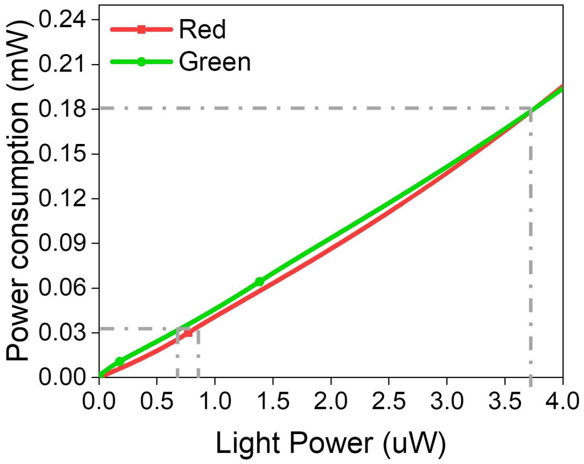

Supplementary Figure 5. Power consumption-Light power characteristics of FQDLEDs for lifetime measurement.

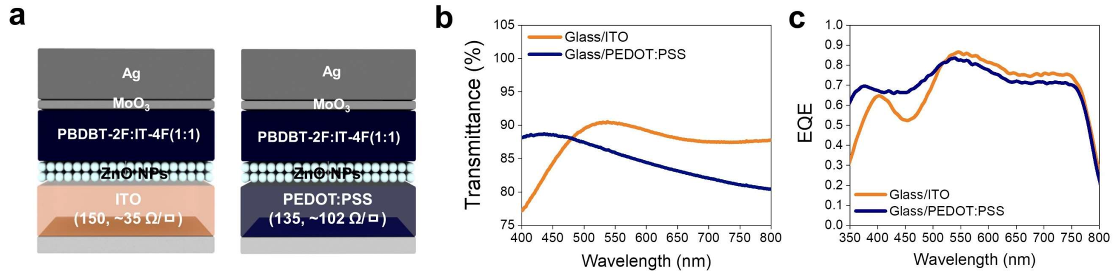

Supplementary Figure 6. OPD characteristics of the ITO and PEDOT:PSS electrodes. (a) Schematic illustration of the OPD structures. **(b,c)** Transmittance and external quantum efficiency (EQE) by wavelength.

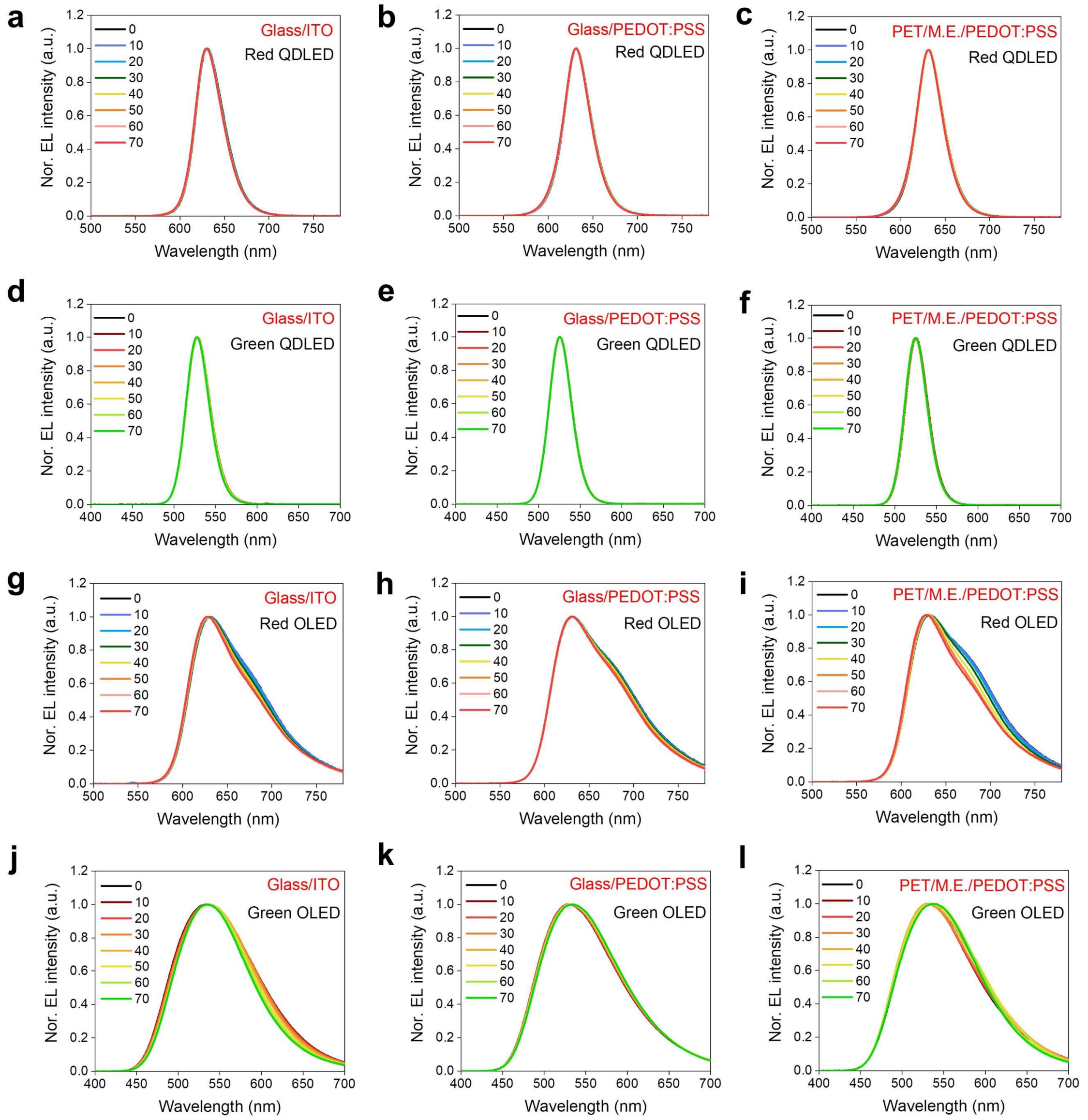

Supplementary Figure 7. Angular spectral characteristics for the three different structures (Glass/ITO, Glass/PEDOT:PSS, PET/ME/PEDOT:PSS) of the QDLEDs and OLEDs. (a,b,c) Red QDLEDs. **(d,e,f)** Green QDLEDs. **(g,h,i)** Red OLEDs. **(j,k,l)** Green OLEDs.

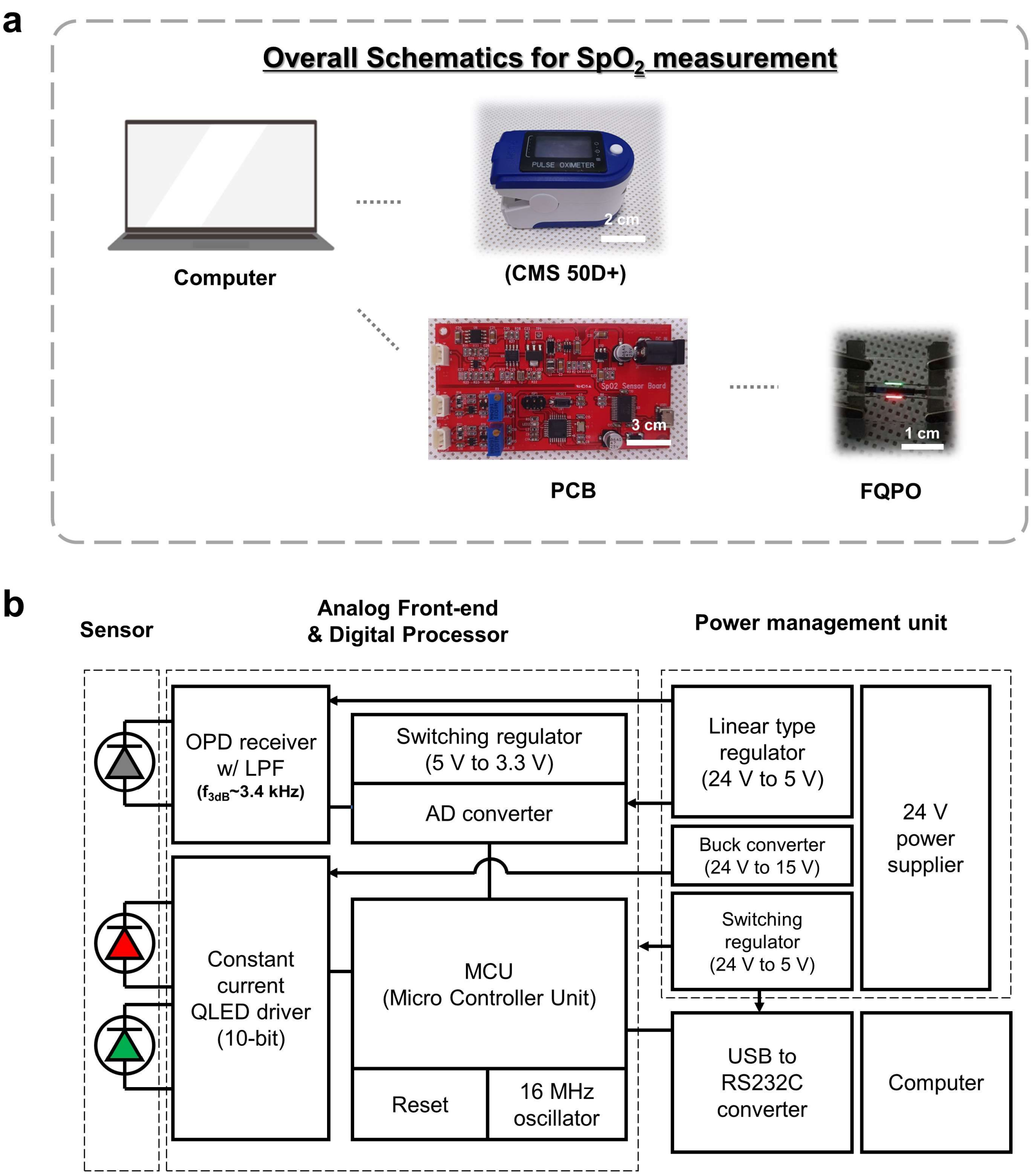

Supplementary Figure 8. System for measuring oxygen saturation. (a) Overall schematics for SpO_2 monitoring. **(b)** Schematic diagram of planar circuit board (PCB).

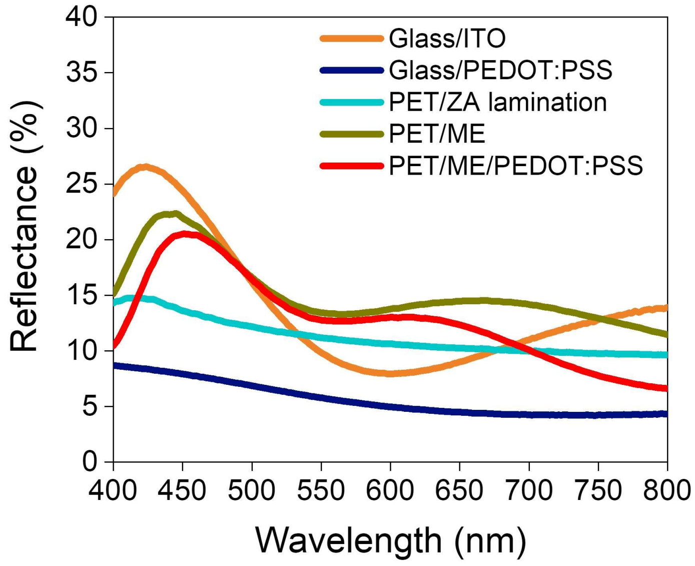

Supplementary Figure 9. Reflectance characteristics according to the layer structure.

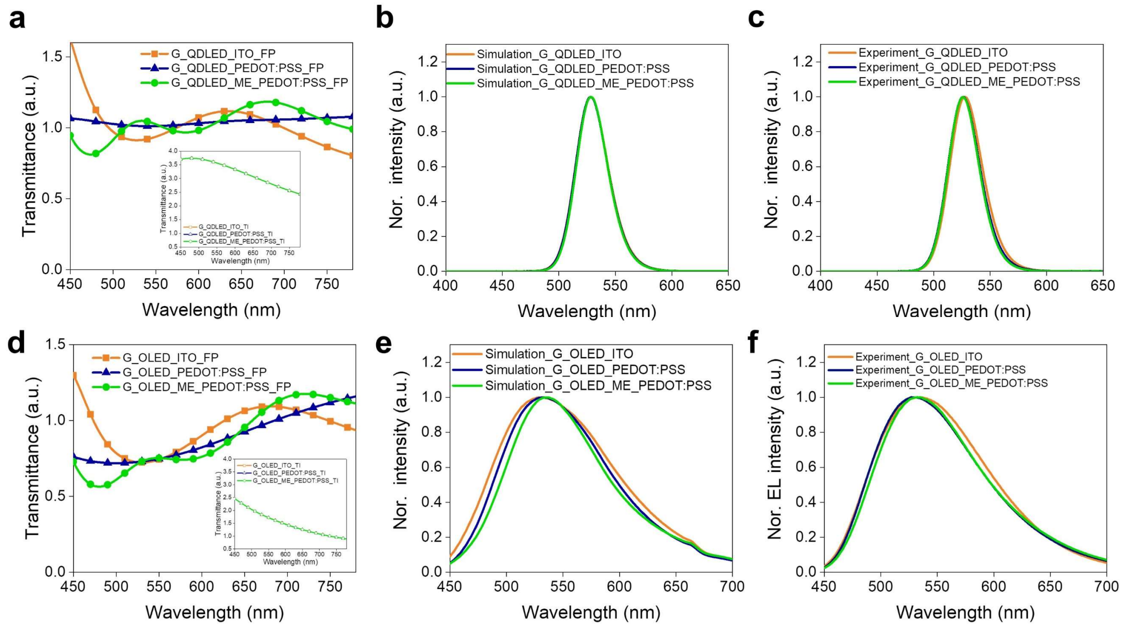

Supplementary Figure 10. Simulation and experiment result of the cavity effect based on green QDLEDs and OLEDs. (a,b,d,e) Simulation results for the FR and TI characteristics and spectral characteristics. **(c,f)** Spectral characteristics from experiment results.

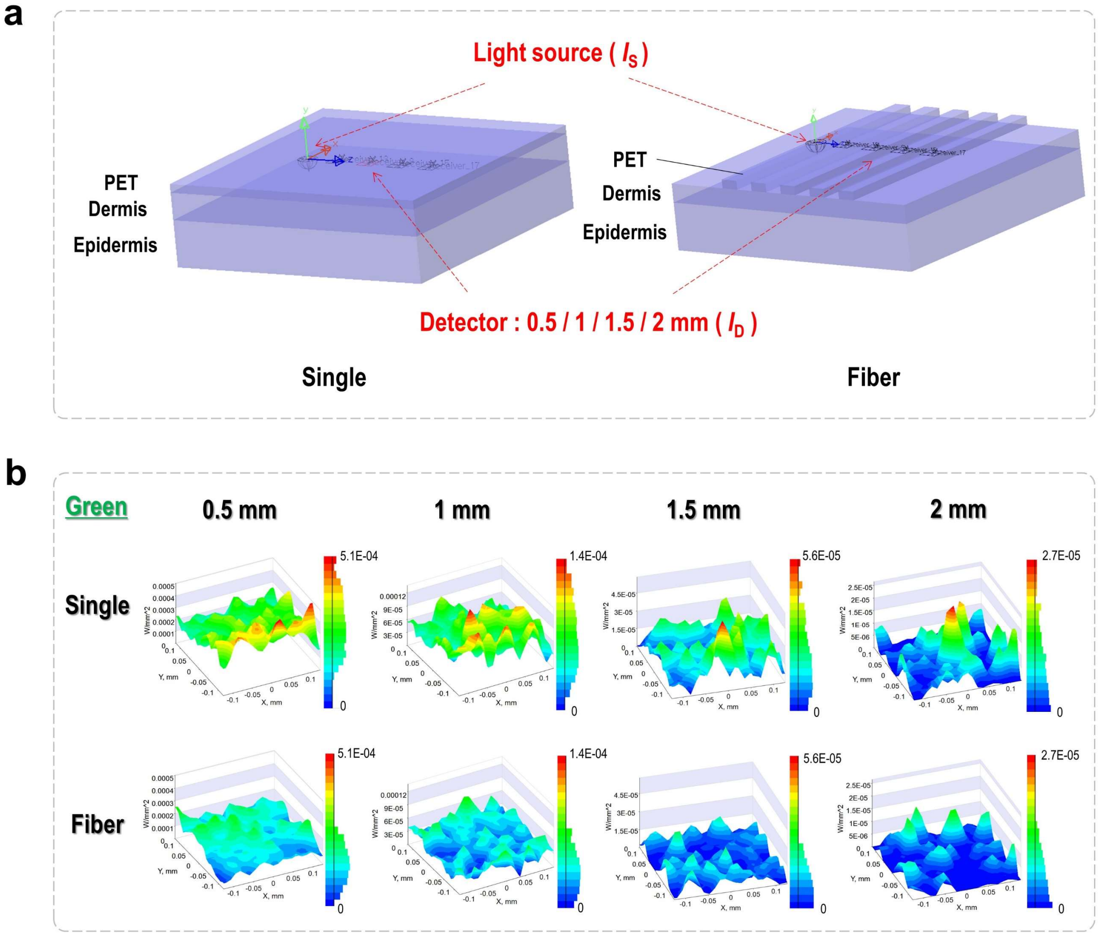

Supplementary Figure 11. Ray tracing simulation result for the waveguide mode ratio of the single and fiber substrates. (a) Schematic illustration of the simulation design. **(b)** Comparison of radiance by distance in single and fiber substrates of the green light sources (Area: 0.2 X 0.2 mm^2).

Supplementary Tables

Supplementary Table 1. Thickness and sheet resistance characteristics depending on the spin-coating speed of the PEDOT:PSS electrode.

Spin speed	Thickness (nm)	Sheet resistance (Ω/□)
800 rpm	160	84 ± 2.15
1000 rpm	135	99.86 ± 3.24
1400 rpm	100	118.4 ± 2.7

Supplementary Table 2. External quantum efficiency and responsivity characteristics according to electrodes in red and green wavelength bands.

	Red (λ = 630 nm)		Green (λ = 530 nm)	
TCE	ITO	PEDOT:PSS	ITO	PEDOT:PSS
EQE (%)	77.8	73.2	84.8	83.2
Responsivity (A/W)	0.395	0.372	0.363	0.356

Supplementary Table 3. The number of incident rays to detector by substrate, color and distance among total 100,000 rays.

The number of total ray = 100,000		Distance from light source to detector			
		0.5 mm	1 mm	1.5 mm	2 mm
Single	Red	2666	804	282	144
	Green	2762	869	334	133
Fiber	Red	1516	404	202	73
	Green	1647	502	186	73
